# XeroGraph: enhancing data integrity in the presence of missing values with statistical and predictive analysis

**DOI:** 10.1093/bioadv/vbaf035

**Published:** 2025-02-21

**Authors:** Laila Mousafi Alasal, Emma U Hammarlund, Kenneth J Pienta, Lars Rönnstrand, Julhash U Kazi

**Affiliations:** Division of Translational Cancer Research, Department of Laboratory Medicine, Lund University, Lund, 22363, Sweden; Lund Stem Cell Center, Department of Laboratory Medicine, Lund University, Lund, 22184, Sweden; Lund University Cancer Centre (LUCC), Lund University, Lund, 22184, Sweden; Lund Stem Cell Center, Department of Laboratory Medicine, Lund University, Lund, 22184, Sweden; Lund University Cancer Centre (LUCC), Lund University, Lund, 22184, Sweden; Tissue Development and Evolution (TiDE), Department of Experimental Medical Science, Lund University, Lund, 22184, Sweden; The Cancer Ecology Center, Brady Urological Institute, Johns Hopkins School of Medicine, Baltimore, MD, 21287, United States; Division of Translational Cancer Research, Department of Laboratory Medicine, Lund University, Lund, 22363, Sweden; Lund Stem Cell Center, Department of Laboratory Medicine, Lund University, Lund, 22184, Sweden; Lund University Cancer Centre (LUCC), Lund University, Lund, 22184, Sweden; Division of Hematology, Oncology and Radiation Physics, Skåne University Hospital, Lund, 22242, Sweden; Division of Translational Cancer Research, Department of Laboratory Medicine, Lund University, Lund, 22363, Sweden; Lund Stem Cell Center, Department of Laboratory Medicine, Lund University, Lund, 22184, Sweden; Lund University Cancer Centre (LUCC), Lund University, Lund, 22184, Sweden

## Abstract

**Motivation:**

Missing data present a pervasive challenge in data analysis, potentially biasing outcomes and undermining conclusions if not addressed properly. Missing data are commonly classified into Missing Completely at Random (MCAR), Missing at Random (MAR), and Missing Not at Random (MNAR). While MCAR poses a minimal risk of data distortion, both MAR and MNAR can seriously affect the results of subsequent analyses. Therefore, it is important to know the type of missing data and appropriately handle them.

**Results:**

To facilitate efficient handling of missing data, we introduce a Python package named XeroGraph that is designed to evaluate data quality, categorize the nature of missingness, and guide imputation decisions. By comparing how various imputation methods influence underlying distributions, XeroGraph provides a systematic framework that supports more accurate and transparent analyses. Through its comprehensive preliminary assessments and user-friendly interface, this package facilitates the selection of optimal strategies tailored to the specific missing data mechanisms present in a dataset. In doing so, XeroGraph may significantly improve the validity and reproducibility of research findings, making it a valuable tool for professionals in data-intensive fields.

**Availability and implementation:**

XeroGraph is compatible with all operating systems and requires Python version 3.9 or higher. It can be freely downloaded from PyPI (https://pypi.org/project/XeroGraph). The source code is accessible on GitHub (https://github.com/kazilab/XeroGraph), and comprehensive documentation is available at Read the Docs (https://xerograph.readthedocs.io). This software is distributed under the Apache License 2.0.

## 1 Introduction

In the context of data science, the quality and completeness of data are critical factors that dictate the accuracy and reliability of analysis (Wang *et al.* 2024). Missing values in datasets pose a significant challenge, as they can obscure underlying patterns and potentially lead to biased or incorrect conclusions. Missing values can occur due to a variety of reasons, such as errors in data collection, failure in data recording processes, or intentional omission (Fouad *et al.* 2021). The impact of missing data is not merely a technical inconvenience but a substantial barrier that can compromise the integrity of statistical inference and predictive modeling.

The complexity of addressing missing data lies in understanding the nature of its occurrence and the statistical ramifications of different imputation strategies. Missing data or missingness can be classified into three categories: Missing Completely at Random (MCAR), Missing at Random (MAR), and Missing Not at Random (MNAR) (Lee *et al.* 2023). Missingness categorized as MCAR occurs when the probability of a data point being missing is entirely independent of both observed and unobserved data. This type of missingness arises purely by chance, reflecting randomness in the data collection process rather than any systematic relationship with the variables themselves. Therefore, statistical methods applied to datasets with MCAR remain unbiased. While missingness in MAR depends on observed data, it is independent of unobserved data. This means that imputations can leverage relationships between variables for unbiased estimates when modeled correctly. MNAR is categorized as the missingness related to the value of the missing data itself. Accurately imputing MNAR data often requires external assumptions or additional data, such as prior knowledge about the mechanism of missingness, access to related variables that can act as proxies or external datasets that provide complementary insights into the missingness pattern.

The distinctions among these types of missing data are crucial for selecting the appropriate strategies for handling missing data. While MCAR is conceptually straightforward and relatively easy to reproduce artificially in datasets, MAR and MNAR require careful consideration and context-specific understanding. Inappropriate handling of missing data can lead to various issues, such as bias, reduced efficiency, and even invalidation of the research results (Pedersen *et al.* 2017).

MAR occurs when the probability of a value being missing is related to observed information but not to the missing value itself. In other words, once we account for the variables we do observe, the missingness no longer depends on unobserved values. For example, in a large health survey, participants are asked both demographic questions (e.g. age, gender, education) and behavioral questions (e.g. diet, exercise frequency). Suppose younger respondents (a fully observed characteristic) are slightly less likely to report their annual income. If we know each participant’s age group, gender, and education level, the probability of income being missing can be explained by these observed factors. Thus, once these demographic factors are accounted for, the missingness in income does not depend on the actual unreported income value itself. In this scenario, the missingness in income is MAR. Another example can be in a standardized testing setting; some students skip certain test questions. These omissions may be associated with characteristics like their academic background or the difficulty rating of the question (both known quantities). For instance, lower-performing students—identified by their previously observed scores—might be more inclined to leave complex math problems unanswered. Once we control for these observable performance metrics, the missingness on those specific test items can be treated as MAR because the likelihood of missing an answer depends on known variables (previous scores, item difficulty), not on the student’s “true” but unobserved ability on that specific question. In other words, the missing values are tied to the observed outcome.

On the other hand, MNAR occurs when the probability of a value being missing is related to the value that is missing itself. In other words, even after considering all observed data, the fact that some data are missing is inherently tied to what those missing values would have been if observed. For example, in a clinical drug trial, patients are asked to return for follow-up visits to measure a key outcome (e.g. blood pressure or a biomarker level). If the sickest patients—those whose outcomes would be the worst—are more likely to drop out or fail to return for follow-up appointments because of their declining health, then the missingness is driven by the values themselves. The participants’ missing data on health measurements are not fully explained by their initial characteristics or other observed factors. Instead, the missing values are tied to the unobserved outcome: those with worse outcomes are disproportionately missing. This situation often leads to MNAR. Furthermore, we may consider a survey on personal spending habits within a pool of individuals. Wealthier individuals might be less inclined to disclose their exact spending on luxury items due to privacy concerns. If the reason they skip these questions is inherently linked to their higher spending (i.e. the missingness depends directly on the true, unreported value), then the data are MNAR. Even if you know their incomes and demographic profiles, these known variables may not fully explain why they specifically opted out of reporting luxury spending. The probability of missingness still depends on the very value that is missing.

To characterize and overcome these challenges, we introduce XeroGraph, a comprehensive Python package designed to assist in the management of missing data. XeroGraph offers tools for assessing data quality, identifying the type of missingness, and implementing advanced imputation methods tailored to the specific conditions of the dataset. XeroGraph not only identifies and imputes missing data but also analyzes the impact of different imputation techniques on data distribution. By enhancing the handling of missing values, XeroGraph aims to bolster the reliability of data analyses, ensuring that conclusions drawn from incomplete datasets are both valid and verifiable.

## 2 Implementation

XeroGraph is a comprehensive Python library designed to address the complexities associated with missing data in datasets. It equips users with robust analytical functions, a diverse suite of imputation methods, and tools for evaluating and comparing imputation performance. Here, we provide the statistical rationale, methodologies, and motivations underlying its design.

### 2.1 Analytical functions

Effective handling of missing data begins with a thorough understanding of the dataset. XeroGraph provides seven analytical functions to aid this process as described below.

#### 2.1.1 Normality tests

Many statistical models assume normally distributed data. XeroGraph includes the Shapiro-Wilk test, which evaluates whether a dataset follows a normal distribution (Shapiro and Wilk 1965). XeroGraph utilized “shapiro” function from SciPy that calculates W statistics using the following Equation (1) and *P*-value for the hypothesis test.

(1) $$W=\frac{{\left(\sum_{i=1}^{n}a_{i}x_{i}\right)}^{2}}{\sum_{i=1}^{n}{\left(x_{i}-\bar{x}\right)}^{2}}\;$$

Where $x_{i}$: ordered data values sorted from smallest to largest, $\bar{x}$: mean of the sample data, and $a_{i}$: weights based on expected normal order statistics.

If the *P*-value is greater than a chosen significance level (e.g. .05), the data is considered normally distributed; otherwise, it is not. This test is sensitive to deviations from normality, and visual tools like histograms or Q-Q plots are often used alongside it for confirmation.

#### 2.1.2 Kolmogorov-Smirnov test

This non-parametric test compares the empirical cumulative distribution function of observed data $F_{n}(x)$ with a reference distribution F(x), using the statistic $D=\max\left|F_{n}(x)-F_{\left(x\right)}\right|\;$(NLST 2024). It is particularly useful for assessing goodness of fit. The “kstest” function from SciPy was used to calculate *D* statistics and *P*-value against the normal distribution. A smaller *D* value and *P*-value greater than .05 may suggest a particular distribution is a normal distribution.

#### 2.1.3 Visualization tools

XeroGraph includes a number of tools such as histograms, density plots, box plots, and Quantile-Quantile (Q-Q) plots allow for visual examination of data distributions and the detection of outliers or anomalies, which often coincide with missing values.

#### 2.1.4 Missing data patterns visualization

Visualizing missing data patterns using heatmaps or correlation matrices helps identify systematic biases in missingness. For example, a high correlation between missingness in two variables may indicate shared underlying causes.

#### 2.1.5 Visualization of missing percentages

Provides detailed views of the percentage of missing data across features and samples, highlighting areas that may require special attention.

#### 2.1.6 Little’s MCAR test

A pivotal test for determining whether data are MCAR (Little 1988). The test evaluates whether the observed data patterns deviate significantly from what would be expected if the missing data were MCAR. This implementation uses an Expectation-Maximization (EM) algorithm to estimate the mean and covariance matrix of the data under the assumption of multivariate normality (Meng and Van Dyk 1997). To ensure the validity of the test, a Henze-Zirkler multivariate normality check is performed, with a warning provided if the data do not follow a normal distribution. The test outputs a chi-square statistic, degrees of freedom, and a *P*-value, along with detailed information on missingness patterns and their contributions to the result.

#### 2.1.7 Combined MCAR and MAR-MNAR test

This function integrates Little’s MCAR Test with a likelihood-ratio test (LRT) for distinguishing between MAR and MNAR data mechanisms (2). The workflow begins with Little’s MCAR test. If the MCAR hypothesis is rejected, feature-wise MAR-MNAR LRTs are conducted. The MAR-MNAR tests model missingness using logistic regression, where the MAR model predicts missingness as a function of other observed features (3), and the MNAR model adds the missing feature (or an outcome variable) to the MAR model (4). Log-likelihoods are computed for both models, and the difference is assessed using a LRT. A significant result suggests that missingness is likely MNAR, indicating dependency on the missing feature itself.

log-likelihood:

(2) $$\mathrm{LTR}\;\mathrm{statistics}=2\times \left[l_{\mathrm{MNAR}}-l_{\mathrm{MAR}}\right]$$

MAR model:

(3) $$\log\left(\frac{p(D=1|\mathrm{other}\;\mathrm{features})}{1-p(D=1|\mathrm{other}\;\mathrm{features})}\right)=\beta_{0}+\beta_{1}\left(\mathrm{other}\;\mathrm{features}\right)\;$$

MNAR model:

(4) $$\log\left(\frac{p(D=1|\mathrm{other}\;\mathrm{features},\;Y)}{1-p(D=1|\mathrm{other}\;\mathrm{features},\;Y)}\right)=\beta_{0}^{\prime }+\beta_{1}^{\prime }\left(\mathrm{other}\;\mathrm{features}\right)+\beta_{2}^{\prime }Y\;$$

where *D* is the binary vector indicating whether the feature is observed (1) or missing 0, $\beta_{0}$ is the intercept and $\beta_{i}$ represents the coefficients, $l_{\mathrm{MAR}}$ is the maximized log-likelihood under the MAR model, and $l_{\mathrm{MNAR}}$ is the maximized log-likelihood under the MNAR model.

The implementation handles edge cases such as fully observed or fully missing features, small sample sizes, and datasets with only one variable. It includes a parameterized significance level (α) for hypothesis testing and automatically adjusts for datasets with or without an outcome variable (*Y*). The output includes results from Little’s MCAR test (chi-square statistic, degrees of freedom, *P*-value, conclusion, and comment) and feature-specific MAR-MNAR results (LRT statistic, *P*-value, and conclusion: MAR or MNAR). This combined framework offers a robust and interpretable approach to identifying the underlying mechanism of missing data, providing essential insights for downstream analysis.

### 2.2 Missing value imputation methods

XeroGraph includes a diverse array of imputation methods, each suitable for different types of data and missingness mechanisms. We present eight of these methods below.

#### 2.2.1 Simple imputers

This method includes mean, median, and mode (most frequent) imputations which are quick and effective for uniformly missing data (Pedregosa *et al.* 2011). However, they assume data are MCAR and risk underestimating variance.

#### 2.2.2 KNN imputation

Utilizes the k-nearest neighbors algorithm to predict missing values based on the similarity of instances (Pedregosa *et al.* 2011). This method may be effective for MAR data with local dependencies.

#### 2.2.3 Iterative imputation

Models each feature with missing values as a function of other features, employing a round-robin approach (Pedregosa *et al.* 2011). This approach converges to stable estimates.

#### 2.2.4 Random forest imputation

Uses a random forest model to predict missing values along with iterative imputation, adept at handling non-linear relationships within complex data structures (Pedregosa *et al.* 2011). The iterative process refines predictions by leveraging non-linear relationships within the data.

#### 2.2.5 Lasso CV imputation

Implements Lasso regression with cross-validation within iterative imputation to estimate missing values, especially useful in preventing overfitting (Pedregosa *et al.* 2011).

#### 2.2.6 XGBoost imputation

Takes advantage of the robust XGBoost algorithm with iterative imputation to address missing values, particularly effective in large and complex datasets (Chen and Guestrin 2016). This method optimizes missing value predictions using loss functions like mean squared error (MSE).

#### 2.2.7 Xputer

A recently developed method that integrates matrix factorization with XGBoost for enhanced prediction accuracy in missing value imputation (Younus *et al.* 2024).

#### 2.2.8 MICE imputation

The Multiple Imputation by Chained Equations (MICE) technique iteratively performs multiple imputations to fill in missing values, accommodating various patterns of missing data (van Buuren and Groothuis-Oudshoorn 2011). Each imputed dataset is analyzed independently, and results are pooled for inference. This process is computationally expensive and can take a very long time for a larger dataset.

### 2.3 Imputation quality analysis functions

The imputation quality analysis functions in XenoGraph offer comprehensive tools to evaluate the plausibility and reliability of imputed data using both statistical and visual analyses. These functions are designed to check whether the imputed values are within the range of the observed data. Specifically, XenoGraph provides feature-wise summary statistics to evaluate the overall data integrity, visual assessments of distributions through density plots and box plots, and analyses of data dispersion to identify potential anomalies. Additionally, the tool includes functionality to statistically evaluate whether significant differences exist between the observed and imputed data, informing that the imputation process does not introduce biases that could affect subsequent interpretations. These functions provide an overview of the overall imputation quality. By integrating statistical checks and visual assessments, the tool allows users to evaluate imputed values, ensuring that the imputation process aligns with the original data patterns and meets the demands of data analysis. This focus on quality assessment enhances user confidence in the results.

### 2.4 Comparative analysis function

XeroGraph can compare different imputation methods. This function facilitates the selection of the most appropriate imputation model based on performance metrics tailored to the specific characteristics of the data. Users can evaluate the effectiveness of each method in handling the peculiarities of their dataset, leading to more informed decisions about which imputation technique to deploy. It introduces 10%–20% missing values randomly and then calculates RMSE and *P*-value after imputation.

## 3 Usage in data analysis

The XeroGraph library provides a straightforward workflow, enabling users to analyze and manage missing data through intuitive steps. The process involves initializing the library with the dataset, performing exploratory data analysis, applying imputation methods, and evaluating the results. Comprehensive documentation and examples are available at XeroGraph Documentation (https://xerograph.readthedocs.io).

### 3.1 Initializing the XeroGraph analyzer (XeroAnalyzer)

To begin, users initialize the XeroGraph analyzer with their dataset. This step creates an instance of the XeroAnalyzer object, which serves as the central point for data exploration and imputation. During initialization, users can configure settings such as whether an outcome feature is available to save output visualizations and specify the directory path for saved plots. This flexibility allows users to customize the workflow according to their needs and maintain organized records of the analysis.

### 3.2 Performing data analysis

**Figure 1.**
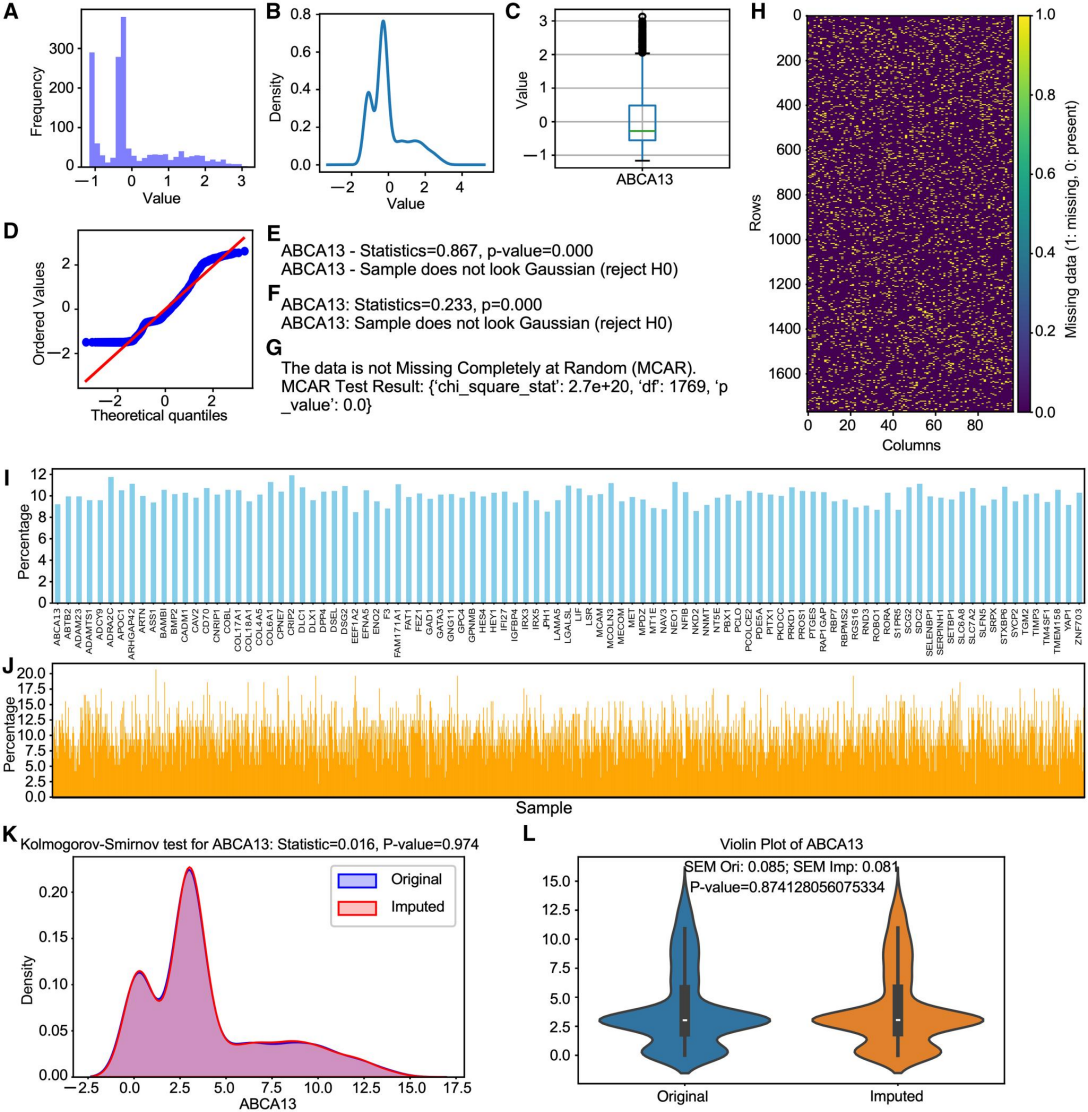
Examples of XeroGraph outputs. The figure shows the XeroAnalyzer outputs, including a histogram (A), density plot (B), box plot (C), Q-Q plot (D), normality test output (E), Kolmogorov-Smirnov test output (F), Little’s MCAR test (G), patterns of missing data (H), percentages of missing values feature-wise (I), percentages of missing values sample-wise (J), overlapping density plots for original and imputed data (K), and violin plots for original and imputed data (L).

XeroGraph offers statistical tests and visualization tools for comprehensive data analysis. By invoking specific methods, visualizations including histograms (Fig. 1A), density plots (Fig. 1B), box plots (Fig. 1C), Q-Q plots (Fig. 1D), normality tests (Fig. 1E), the Kolmogorov-Smirnov test (Fig. 1F), Little’s MCAR test (Fig. 1G), analysis of missing data patterns (Fig. 1H), and calculation of missing data percentages across features (Fig. 1I) and samples (Fig. 1J) can be generated. Additionally, XeroGraph provides a brief missing type analysis method that checks whether missing values fall into MCAR, MAR, or MNAR types. The method has been tested on both real-life and artificially constructed datasets to evaluate its ability to classify and analyze missingness patterns effectively (Table 1). For real-life data, we employed the AIDS Clinical Trials Group Study 175 (ACTG175) dataset (https://www.rdocumentation.org/packages/speff2trial/versions/1.0.5/topics/ACTG175), which contains 27 features, with one feature exhibiting ∼37% missing values. Using XeroGraph’s missing-type analysis, we identified this highly missing feature in ACTG175 as likely belonging to the MNAR category. To further evaluate XeroGraph’s performance, we constructed multiple artificial datasets with explicitly defined missingness mechanisms. We generated three distinct simulated datasets—Simulated_data_mcar, Simulated_data_mar, and Simulated_data_mnar—by systematically introducing missing values based on MCAR, MAR, and MNAR mechanisms, respectively. The automated classification results closely aligned with the intended underlying patterns, confirming the accuracy of XeroGraph's detection capabilities. Additionally, four real-world datasets (AML, Breast_cancer, PIMA_Indian, and Student_dropout) were used to introduce random missing values for further validation. All datasets are readily accessible through XeroGraph’s “load_dataset” function.

**Table 1.** Missing type analysis.

Dataset	Total missing (%)	Number of features	Features with missing data	MCAR/normality test	MAR positive features	MNAR positive features
Example dataset	4.0	5	4	Positive/failed	3	1
ACTG175	1.4	26	1	Negative/failed	0	1
AML	10.0	97	97	Negative/failed	0	97
Breast cancer	10.2	30	30	Negative/failed	17	13
PIMA Indian	9.8	8	8	Positive/failed	0	8
Student’s dropout	9.9	36	36	Negative/failed	8	28
Simulated data for MCAR	10.0	5	5	Positive/passed	Not performed	Not performed
Simulated data for MAR	29.8	10	10	Negative/failed	8	2
Simulated data for MNAR	30.3	10	10	Negative/failed	10	10

### 3.3 Performing imputation

The library provides several imputation methods tailored to different types of data and missingness mechanisms, from basic to advanced methods. Each of these ten different methods can be individually applied by calling specific functions on the initialized data.

### 3.4 Post-imputation analysis

After conducting imputations, evaluating the quality and plausibility of the imputed data is crucial. XeroGraph enables the application of two different methods for this purpose: plausibility checks (Fig. 1K) and comparative statistical analysis (Fig. 1L).

### 3.5 Comparing imputation methods

To facilitate informed decisions about the best imputation techniques, XeroGraph includes the XeroCompare functionality. This method can be applied to initialized data or used separately. It allows for a thorough comparison of various imputation methods, providing insights based on performance metrics tailored to the specific data (Supplementary Table S1).

## 4 Conclusion

XeroGraph is a Python tool for researchers, data scientists, and analysts for handling missing data in the datasets. Offering a comprehensive suite of analytical functions and a diverse array of imputation methods, XeroGraph provides a framework for data analysis and enhancement. The library’s capabilities span from basic data visualizations and statistical tests to advanced imputation techniques, addressing missingness types, including MCAR, MAR, and MNAR, which are critical for accurate data handling.

The design of XeroGraph is user-friendly, ensuring that users can effectively implement and benefit from its features, regardless of their coding expertise. The library provides tools for analyzing patterns of missing data and filling these gaps with statistically sound methods, thereby facilitating reliable data analysis. Additionally, the inclusion of comparative analysis functions enables users to make informed decisions based on observed indications regarding the suitable imputation method for their specific needs. XeroGraph has been tested with different datasets (Nasimian *et al.* 2024; Younus *et al.* 2024) to verify its performance in detecting missing types (Table 1). Furthermore, a comparative analysis showed that the pre-selection of imputation methods can significantly improve the quality of imputation (Table S1).

Finally, XeroGraph enhances dataset integrity and usability, enabling users to conduct insightful analyses. In a data-driven world, the quality of data directly impacts the insights derived from it. By addressing a common issue in data handling, XeroGraph not only resolves a critical challenge but also advances the field of data analysis. It facilitates improved approaches to data interpretation, which are essential for generating reliable and actionable insights.

## Supplementary Material

vbaf035_Supplementary_Data

## Data Availability

The source code is freely available on GitHub (https://github.com/kazilab/XeroGraph) under the Apache License 2.0. All associated data is openly accessible.
