# Supplementary material for: XeroGraph: enhancing data integrity in the presence of missing values with statistical and predictive analysis
Source: Bioinform Adv. 2025 Feb 21;5(1):vbaf035. doi: 10.1093/bioadv/vbaf035 (PMC11889451; doi:10.1093/bioadv/vbaf035)
Supplement: vbaf035_Supplementary_Data [file vbaf035_supplementary_data.zip › Supplementary table 1.pdf]

Supplementary table 1. Comparing different imputation methods using three datasets. The lowest RMSE values are marked as yellow.

A. Dataset 1

Method	RMSE-mean	RMSE-median	RMSE-min	RMSE-max	P-mean	P-median	P-min	P-max
mean	2.438	2.442	1.561	3.478	0.472	0.448	0.004	0.989
median	2.480	2.468	1.563	3.745	0.177	0.019	0.000	0.996
most_frequent	4.815	4.835	2.340	7.380	0.000	0.000	0.000	0.000
KNN	1.537	1.507	0.840	2.519	0.402	0.338	0.000	0.998
iterative	1.559	1.538	0.971	2.431	0.494	0.500	0.000	0.998
random_forest	1.525	1.506	0.863	2.478	0.488	0.476	0.001	0.993
lasso_cv	1.560	1.551	0.976	2.425	0.493	0.525	0.000	0.968
xgboost	1.569	1.531	0.971	2.518	0.473	0.489	0.000	0.995
xputer	1.465	1.441	0.837	2.393	0.494	0.484	0.000	1.000
mice	2.162	2.122	1.315	3.438	0.454	0.426	0.002	0.981

B. Dataset 2

Method	RMSE-mean	RMSE-median	RMSE-min	RMSE-max	P-mean	P-median	P-min	P-max
mean	32.293	0.080	0.003	473.453	0.422	0.326	0.023	0.964
median	35.338	0.085	0.003	548.698	0.167	0.035	0.000	0.980
most_frequent	45.436	0.136	0.004	715.182	0.033	0.000	0.000	0.975
KNN	12.750	0.063	0.003	163.547	0.390	0.335	0.000	0.982
iterative	3.670	0.026	0.002	43.189	0.461	0.534	0.003	0.999
random_forest	4.675	0.030	0.002	50.926	0.406	0.449	0.010	0.949
lasso_cv	7.739	0.050	0.003	102.699	0.462	0.479	0.001	0.970
xgboost	4.820	0.033	0.001	55.676	0.473	0.492	0.022	0.965
xputer	4.972	0.037	0.002	61.858	0.323	0.141	0.000	0.974
mice	8.353	0.030	0.002	100.681	0.464	0.467	0.043	0.989

C. Dataset 3

Method	RMSE-mean	RMSE-median	RMSE-min	RMSE-max	P-mean	P-median	P-min	P-max
mean	29.134	14.274	0.282	142.570	0.608	0.699	0.128	0.874
median	30.434	14.734	0.300	151.461	0.166	0.021	0.000	0.876
most_frequent	34.868	19.624	0.354	167.566	0.101	0.000	0.000	0.718
KNN	28.683	14.092	0.311	136.046	0.396	0.376	0.007	0.866
iterative	26.254	12.434	0.292	125.014	0.802	0.884	0.274	0.985
random_forest	28.356	12.752	0.307	137.422	0.613	0.616	0.081	0.976
lasso_cv	26.169	12.373	0.295	124.503	0.809	0.900	0.272	0.998
xgboost	29.418	13.523	0.331	142.109	0.548	0.577	0.113	0.938
xputer	28.275	12.691	0.322	139.989	0.558	0.557	0.131	0.961
mice	31.875	17.249	0.472	140.995	0.455	0.419	0.131	0.976
